# The Effect of Mechanically-Generated Vibrations on the Efficacy of Hemodialysis; Assessment of Patients’ Safety: Preliminary Reports

**DOI:** 10.3390/ijerph16040594

**Published:** 2019-02-18

**Authors:** Beata Hornik, Jan Duława, Czesław Marcisz, Wojciech Korchut, Jacek Durmała

**Affiliations:** 1 Department of Internal Nursing, School of Health Sciences in Katowice, Medical University of Silesia, 40-752 Katowice, Poland; 2 Department of Internal Medicine and Metabolic Diseases, School of Health Sciences in Katowice, Medical University of Silesia, 40-752 Katowice, Poland; jdulawa@sum.edu.pl; 3 Department of Gerontology and Geriatric Nursing, School of Health Sciences in Katowice, Medical University of Silesia, 40-752 Katowice, Poland; cmarcisz@sum.edu.pl; 4 University of Social Sciences and Humanities, 40-326 Katowice, Poland; wojciech@korchut.pl; 5 GPE Psychotronics, 43-100 Tychy, Poland; 6 Department of Rehabilitation, School of Health Sciences in Katowice, Medical University of Silesia, 40-752 Katowice, Poland; jdurmala@sum.edu.pl

**Keywords:** hemodialysis, vibrations, physical activity, *Kt*/*V*, urea reduction ratio (URR)

## Abstract

Muscle activity during a hemodialysis procedure improves its efficacy. We have formulated a hypothesis that vibrations generated by a specially-designed dialysis chair can, the same as physical exercise, affect the filtering of various fluids between fluid spaces during the hemodialysis procedure. This prospective and interventional study included 21 dialyzed patients. During a single dialysis session, each patient used a prototype device with the working name “vibrating chair”. The chair’s drive used a low-power cage induction motor, which, along with the worm gear motor, was a part of the low-frequency (3.14 Hz) vibration-generating assembly with an amplitude of 4 mm. Tests and measurements were performed before and after the vibration dialysis. After a single hemodialysis session including five 3-min cycles of vibrations, an increase in Kt/V in relation to non-vibration Kt/V (1.53±0.26 vs. 1.62±0.23) was seen. Urea reduction ratio increased significantly (0.73±0.03 vs. 0.75±0.03). A significant increase in systolic blood pressure was observed between the first and the third measurement (146±18 vs. 156±24). The use of a chair generating low-frequency vibrations increased dialysis adequacy; furthermore, it seems an acceptable and safe alternative to intradialytic exercise.

## 1. Introduction

Dialysis therapy, as a method of renal replacement in end-stage renal disease (ESRD), is based on removal of excessive and toxic metabolic products (uremic toxins) from the intravascular water space [1,2]. One of the obstacles in the efficacy of hemodialysis (HD) is the inefficient exchange of uremic toxins and other filterable substances between extracellular intravascular and extravascular fluid, as well as between extravascular extracellular and extravascular intracellular fluid [3,4]. Previous research has shown that muscle activity during a hemodialysis procedure improves its efficacy [5,6,7]. Despite reports on the effects of vibrations on the skeletal, muscular, and cardiovascular systems [8,9,10,11], the effect of vibrations on hemodialysis remains undetermined.

We have formulated a hypothesis that vibrations generated by a specially-designed dialysis chair can, the same as physical exercise, affect the filtering of various fluids between fluid spaces during the hemodialysis procedure. The use of vibrations in hemodialysis is an innovative experiment and can have a positive influence on the quality of the procedure.

The aim of our research was to assess the impact of low-frequency vibrations generated by a specially-designed dialysis chair on the efficacy and safety of hemodialysis.

## 2. Materials and Methods

This prospective and interventional study included 21 dialyzed patients; the mean age was 58.7±15.9 ($\bar{x}$± SD; age range 27–86 years); 62 percent of these patients were men. The patients were hemodialyzed for 35.2±21.6 months (ranging from 3–84 months) and met the inclusion criteria for the study. All patients were treated at the same dialysis station. The control group comprised the same patients (21 individuals) examined prior to, during, and after the standard dialysis without vibrations. Each patient was provided with a comprehensive explanation regarding the aim of the study, its protocol, and risk and gave their written consent to participate.

The inclusion criteria were as follows: over 18 years of age, hemodialysis treatment for end-stage renal disease of at least 3 months in duration, resting blood pressure below 180/100 mmHg on the day vibrations were to be used, venous glucose range of 100–150 mg/dL, and conscious and voluntary consent to participate.

The exclusion criteria comprised: neoplastic or other systemic disease, cardiological instability (advanced mobility disorders, hypertension resistant to pharmacological treatment, severe arrhythmia difficult to control pharmacologically, New York Heart Association Class IV heart failure, severe heart valve defects), cardiac resynchronization therapy and implantation of a permanent pace maker, potassium levels >6 mmol/L, insufficient vascular access (inability to achieve blood flow in the 250–350 mL/min range, advanced renal osteodystrophy, advanced retinopathy, and advanced musculoskeletal system disorders, as well as refusal to participate in this research.

Temporary contraindications included: hemodynamic instability during the procedure (intradialytic hypotension, weight gain of >6% between hemodialysis procedures).

The study was approved by the Bioethical Committee of the Medical University of Silesia in Katowice (Resolution No. KNW/0022/KB1/54/17) and was carried out according to the Declaration of Helsinki regarding human studies.

### 2.1. Measurements

Detailed medical history was taken with each participant followed by physical examination.

Before and after the vibration dialysis, the following tests and measurements were performed: body weight, body mass index (BMI) calculated, and Charlson Comorbidity Index (CCI) assessed; and the height was measured before the vibration dialysis. Heart rate (HR), systolic blood pressure (SBP), and diastolic blood pressure (DBP) were measured three times (in addition to the five routine dialysis unit BP recordings): right before generating vibrations, after the second vibration cycle, and following completion of vibrations. Fasting blood samples (at least 12 h after the last meal) were taken. Pre-dialysis hemoglobin, albumin, creatinine, phosphorus, parathormone (PTH), sodium, and potassium levels were determined while serum urea concentrations were measured before and after dialysis. The Chronic Kidney Disease Epidemiology Collaboration equation (CKD-EPI) was used to assess the estimated glomerular filtration rate (eGFR) [12].

The following dialysis quality measures were defined:

- The concentration of urea before and after the dialysis, based on which the urea reduction ratio (URR) values were calculated with the formula $\left[1-\left(C_{t}/C_{o}\right)\right]$, where $C_{t}$ is post-dialysis blood urea nitrogen and $C_{o}$ is pre-dialysis blood urea nitrogen [13]
- The dialysis adequacy ratio (Kt/V) calculated using Daugirdas’ equation [14]

The control group (C) included the same patients, but dialysis quality was determined during two consecutive standard dialysis sessions without the vibration chair.

### 2.2. Description of the Intervention

During a single dialysis session, each patient used a prototype device with the working name “vibrating chair”. The chair’s drive used a low-power cage induction motor, which, along with the worm gear motor, was a part of the low-frequency (3.14 Hz) vibration-generating assembly with an amplitude of 4 mm. The motor was powered by a single-phase voltage of 230 V from the voltage inverter.

The device did not pose any danger to the user. The above device met the Polish safety norms (PN-EN ISO 13090-1, PN-ISO 5805). The weight of the device was 55 kg, while the body weight of the patient exposed to vibrations did not exceed 90 kg.

At 90 min following dialysis commencement, the vibration-generating device was turned on for 30 min. Each patient stayed in the chair after the vibration cycle had been completed and the vibration generating device was switched off.

During the 30-min hemodialysis, vibrations were programmed in 3-min intervals, i.e., three minutes of vibration followed by three minutes of regeneration.

The whole intervention consisted of 5 vibration cycles of 3 min each (all vibrations totaled 15 min). Blood pressure measurements and pulse measurements were taken immediately before the first vibration cycle and after the second vibration cycle and the fifth vibration cycle. The hemodialysis procedure with the use of the vibration-generating chair was carried out under strict supervision of the researcher.

### 2.3. Statistics

Statistical analysis was performed using the Statistica 13.1 program (StatSoft, Inc., Tulsa, OK, USA). The results are presented as the means and standard deviations ($\bar{x}$± SD).

Student’s paired *t*-test was used to compare the pre- and post-vibration values of study variables. Measurements at three different time points were compared with ANOVA and Bonferroni post-hoc tests. The level of significance was set at p<0.05.

## 3. Results

Table 1 shows the detailed characteristics of the patients. The most common causes of ESRD were glomerulonephritis (28.5%), diabetic kidney disease (19%), and hypertensive nephropathy (19%). The mean comorbidity index was 4.2±3.1. The majority of the patients had vascular access in the form of an arterio-venous (AV) fistula (81%), and four patients (19%) had a tunneled dialysis catheter. The classic bicarbonate dialysis treatments lasted for 4–4.5 h and were carried out 3–5-times a week using high-flux dialysis membranes. The dialysis fluid flow rate was 500–600 mL/min and blood flow 250–350 mL/min. BMI in the study group was 24.2±3.8 kg/m^2^ ($\bar{x}$ ± SD) (Table 1).

### 3.1. The Influence of Vibrations on Dialysis Quality Measures

**Figure 1:**
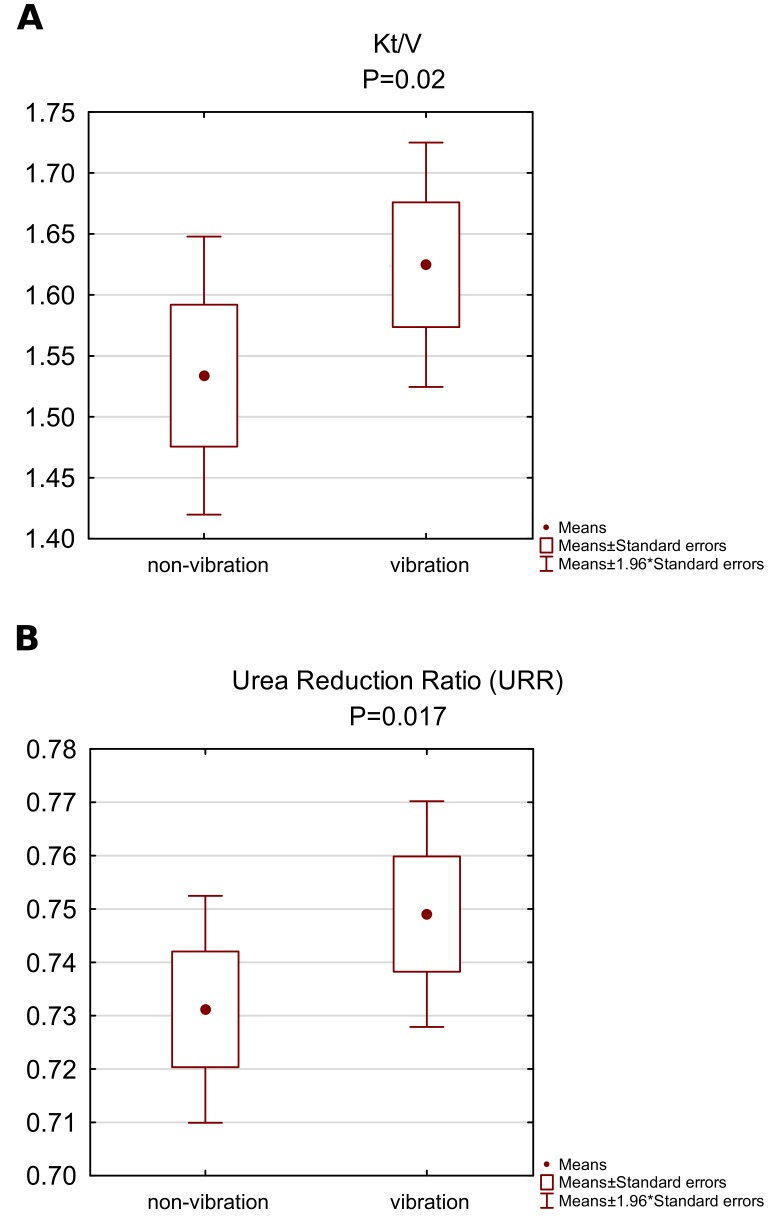
Dialysis adequacy measures of (**A**) Kt/V and (**B**) URR during non-vibration and vibration hemodialysis.

As shown in Figure 1, after a single hemodialysis session including five 3-min cycles of vibrations, an increase in Kt/V in relation to non-vibration Kt/V (1.53±0.26 vs. 1.62±0.23; p=0.02) (Figure 1A) was seen. URR% increased significantly (0.73±0.03 vs. 0.75±0.03; p=0.017) (Figure 1B).

### 3.2. The Effect of Vibrations on Cardiovascular Function Indicators

No adverse cardiovascular events were observed during hemodialysis combined with vibrations. Systolic blood pressure increased insignificantly at the second measurement in comparison to the first and third measurements compared to the second measurement (respectively 146±18 vs. 151±22; p=0.08; 151±22 vs. 156±24; p=0.88). A significant increase in systolic blood pressure was observed between the first and the third measurement (146±18 vs. 156±24; p=0.0002). The three consecutive readings of diastolic blood pressure (86±17; 86±17; 85±18) and heart rate (63 bpm; 65 bpm; 65 bpm) did not show clinically-important or statistically-significant differences. No significant blood pressure or pulse changes were observed in the control group during two consecutive dialyses.

## 4. Discussion

This study is the first to assess the effect of low-frequency vibrations (3.14 Hz) generated by a specially-designed dialysis chair on the quality of dialysis therapy. It was demonstrated that a single dialysis session with the use of low-frequency vibrations improved dialysis adequacy as expressed by Kt/V (1.53±0.26 vs. 1.62±0.23; p=0.02) and URR. The observation may have clinical significance. The dialysis dose based on Kt/V and URR measurements is related to patients’ quality of life and might be considered a factor that influences the morbidity of hemodialyzed patients [15,16,17]. Held et al. [16] analyzed a group of hemodialyzed patients from nearly 350 dialysis centers and demonstrated a decrease in mortality rate by 7% for each 0.1 increase in Kt/V up to a Kt/V of 1.33. Reference ranges of these parameters define the adequate dialysis recommended by international associations. K/DOQI, the National Kidney Foundation/Kidney Disease Outcomes Quality Initiative recommends a Kt/V value of 1.2 and URR ≥ 0.65 for three hemodialyses per week, while the European Best Practice Guidelines recommends Kt/V≥ 1.4 [18,19]. Although some believe that Kt/V is an inadequate measure of adequacy, it seems to be the value most associated with dialysis patients [20]. Numerous studies have indicated that higher Kt/V and URR are linked to better prognosis, although this correlation has not been proven to be causative [21,22]. Chandrashekar et al. [23] concluded that Kt/V was an independent predictor of hemodialysis patient survival. It has been demonstrated that Kt/V≥ 1.4 is associated with a lower mortality risk compared to Kt/V 1.2–1.4 [18]. A number of authors have observed positive correlation between the dose of dialysis and nutrition. Inadequate dialysis (Kt/V< 1) is accompanied by low consumption of protein-rich products [15]. An increase in Kt/V can be achieved by extending the time of a dialysis session, increasing the frequency of dialysis sessions [24], or through intradialytic exercise [25]. Hemodialysis patients are characterized by insufficient physical activity. One of the causes is immobility during dialysis, which totals to approximately 500–800 h a year, as well as during commuting to the dialysis station, which takes around 400–500 h yearly [26]. Numerous reports indicate that exercising during hemodialysis has a positive effect on the quality of life, cytokine concentrations, and hemodialysis quality [27,28,29,30]. There are various methods of implementing exercises during a hemodialysis procedure, i.e., bicycle ergometer exercises under the supervision of a physiotherapist, relaxation exercises, or a combination of several techniques. However, patient participation in intradialytic rehabilitation is limited [31] due to barriers to physical activity during hemodialysis [32]. The most common ones include: fatigue, fear of moving during the procedure, or lack of motivation. There is also reluctance on the part of the medical personnel and organizational barriers.

An alternative to physical exercise programs was introduced by Farese et al. [33]. They found that both intradialytic electrostimulation of thigh and calf muscles and a passive workout on a bicycle ergometer affected the efficiency of dialysis, i.e., urea and phosphate removal were increased, and so was blood pressure. Urea reduction reported by Farese et al. was comparable to the effect achieved using a vibration-generating dialysis chair.

Experimental results published in recent years have confirmed that exposure to carefully-selected vibration parameters (frequency and amplitude of vibrations) may positively affect the cardiovascular system, as well as skeletal and muscle tissues [34].

On the other hand, there are also reports documenting the negative impact of vibrations on the human body [35].

Vibrations at frequencies exceeding the sensitivity threshold may cause pain. The strongest sensation of vibrations occurs at frequencies of up to 35 Hz, especially in the 20-Hz range. Still, the majority of studies using vibrations did not reveal serious side effects [36]. One of the first studies on vibrations assessed their impact on reducing the negative effects of immobility. A group of healthy men who spent five weeks in an oscillating bed retained greater motor fitness and had a lower risk of osteoporosis than individuals in a standard hospital bed [37]. Previous studies indicated a positive effect of vibrations on muscular isometric strength and balance control. An increase in mineral density in the femoral neck was also observed [38].

There is little research available on the application of vibrations in hemodialyzed patients. Seefried et al. [39] noted the benefits of employing a vibration platform for 8–12 weeks before or during dialysis, twice a week at three levels of difficulty. Exercise duration increased gradually from 5–20 min, and the frequency ranged from 5–28 Hz. The amplitude was increased from 0.5–3 mm in the final weeks of the experiment, depending on exercise difficulty. The authors observed that changes in the concentrations of urea did not reach the level of statistical significance. The greatest improvement was found in the muscle strengthening, inflammation parameters, and the cardiovascular system [39]. In the study of Doyle et al., eight weeks of whole-body vibration did not result in a significant change in URR compared to pre-intervention values [40]. In contrast to the above results, our study revealed a significant increase in URR. This discrepancy may have stemmed from differences in the intervention protocols; Doyle et al. used a vibrating platform for three minutes before each dialysis session, while we used intradialytic vibrations. Furthermore, Doyle et al. used a higher frequency and amplitude than we did (50 vs. 3.14 Hz; 10 vs. 4 mm). Sitja-Rabert et al. conducted a systematic review of the literature on whole-body vibration programs in older populations and a meta-analysis of randomized controlled clinical trials [41]. Different vibrations schemes were used in older, not dialyzed patients. The frequency of vibration ranging from 10–40 Hz and amplitude ranging from 0.7–8 mm were used [42,43].

Those randomized control tests assessed the efficacy and safety of whole-body vibration training in older populations in comparison with conventional exercise groups or control groups not performing any exercises. Balance, muscle strength, falls, bone mineral density, as well as adverse events were evaluated [42,44].

**Figure 2:**
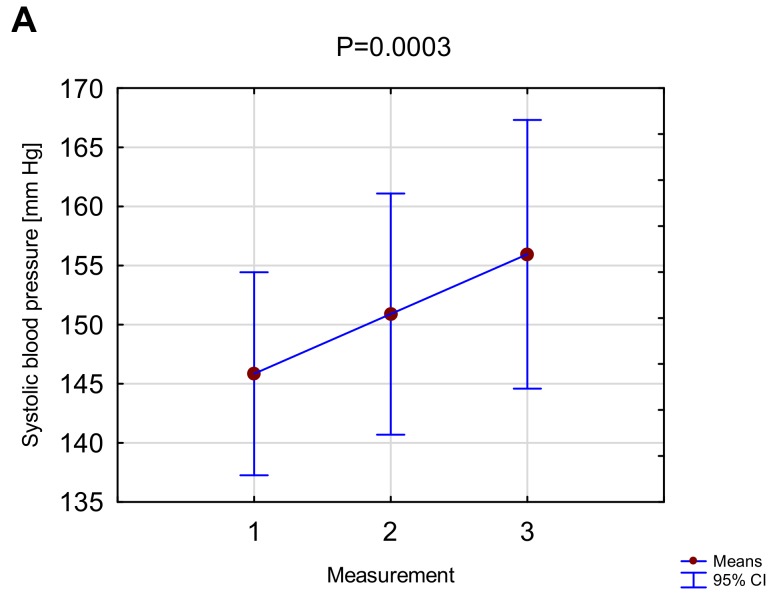

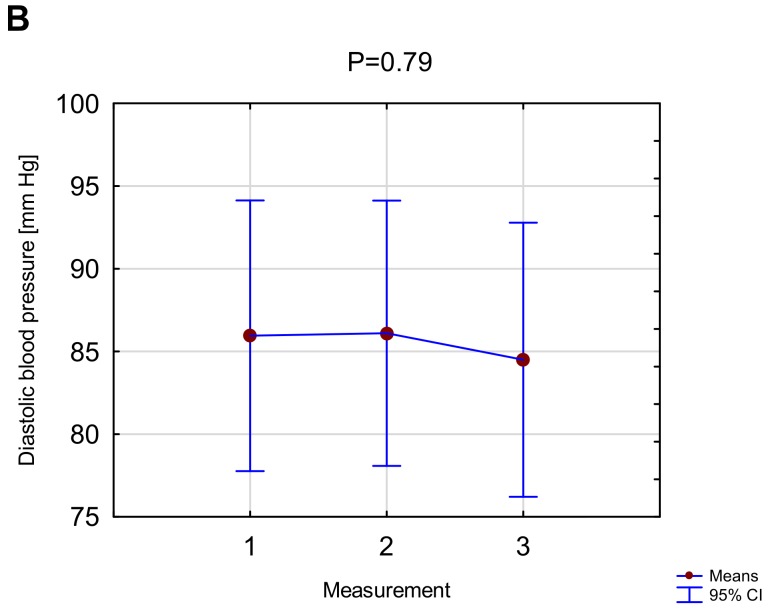
Systolic (**A**) and diastolic (**B**) blood pressure values in three consecutive measurements carried out during vibration hemodialysis. 1, baseline measurement before the first cycle of vibrations; 2, measurement after the second cycle of vibrations; 3, final measurement after the fifth cycle of vibrations.

In addition to dialysis quality, we assessed the effect of vibrations on basic hemodynamic parameters. A significant increase in systolic blood pressure after three cycles of vibrations was found in relation to the baseline values (Figure 2A), while no significant change was revealed in diastolic blood pressure and heart rate (Figure 2B). Such blood pressure response is comparable to hemodynamic response during exercise with increased loads [45].

The effect of regular physical activity on lowering blood pressure has been well established, and the exaggerated response of blood pressure, especially systolic blood pressure, to exercise may be reduced by regular exercise [46]. Maintaining normal blood pressure during hemodialysis and preventing intradialytic hypotension, in particular, is of key importance for the safety of patients [47]. Hemodynamic response to intradialytic whole-body vibrations seems to be similar to the response to intradialytic exercise. Due to the lack of studies assessing cardiovascular risk during hemodialysis combined with vibrations, we were apprehensive about intradialytic hypotension episodes in the studied group. Research conducted by Madhavan et al. indicated that vibrations would effectively suppress the negative effects of a prolonged semi-recumbent position, simulating the effect of a venous pump characteristic of walking, as well as increase mean blood pressure [48]. The effects of vibrations on the body in contrast to traditional resistance exercise remain unclear [49]. Intradialytic hypotension during hemodialysis combined with vibrations was not observed in our study.

Based on our results, we put forward a cautious hypothesis that the transient effect of a small increase in systolic blood pressure brought on by vibrations could help prevent intradialytic hypotension, the most frequent acute complication in hemodialysis [50].

Exposure to low-frequency vibrations (3.14 Hz) may be the equivalent to intradialytic exercise, including resistance training, and can be used during hemodialysis. The increase in Kt/V observed after vibrations was probably caused by an increase of urea redistribution from other compartments into the plasma compartment. Other probable mechanisms should also be considered. One such mechanism is the possible reduction of recirculation within vascular access. Vascular access has been subjected to the same vibrations as the entire body and could indeed reduce the recirculation around the vascular access, as well as the Kt/V increase.

It should also be noted that no problems with vascular access were observed during dialysis combined with vibrations. There were no other vibrations, and the lines were properly anchored. Mueller et al. have noted that urea clearance was increased at transverse vibrations of a high frequency hemodialyzer (120 Hz frequency). According to the quoted authors, dialyzer vibrations of lower frequencies did not increase the clearance [51]. Our research utilized a much lower frequency (3.14 Hz), and the hemodialyzer was stabilized well. It was not possible for the vibrations generated by our “dialysis chair” to cause the vibration effect in the hemodialyzer.

The study has several limitations. Firstly, it presents the effect of arbitrarily-defined vibration intensity during a single hemodialysis session. Other vibration schemes might induce different effects. Secondly, patients who qualified for hemodialysis using vibrations were less burdened by cardiovascular problems, which are associated with a lower risk of complications. Thirdly, due to the small sample size, our findings should be interpreted with caution.

Nonetheless, our study can be considered a starting point for further research of longer duration and in a larger group to verify the effect of vibrations on Kt/V and hemodynamic balance during hemodialysis.

## 5. Conclusions

The use of a chair generating low-frequency vibrations increased dialysis adequacy as measured by Kt/V; furthermore, it seems to be an acceptable and safe alternative to intradialytic exercise.

## Figures and Tables

**Table 1:** Baseline characteristics of the patients participating in the study (n=21).

Variables	Study Group (HD) n=21	
Age (years)	58.7±15.9	(range = 27–86)
Sex, female/male, *n* (%)	8 (38)/13 (62)
Dialysis vintage (months)	35.2±21.6	(range = 3–84)
Duration of dialysis (min)	247±10	(range = 240–270)
Weekly dialysis time (min)	749±68	(range = 630–980)
Dialysis adequacy ($Kt/V_{\mathrm{Daugirdas}}$)	1.62±0.23	(range = 1.28–2.07)
URR	0.74±0.05	(range = 0.67–0.86)
Dialysis fluid flow (mL/min)	505±22	(range = 500–600)
BFR during dialysis (mL/min)	300±13	(range = 280–330)
Temperature of the dialysis fluid (°C)	36.4±0.6	(range = 35.0–37.0)
BMI (kg/m^2^)	24.2±3.8	(range = 19.6–30.8)
Target body weight (kg)	68.1±20.1	(range = 52–90)
Residual renal function (mL)	842±645	(range = 0–2000)
nPCR (g/kg per day)	1.04±0.2	(range = 0.6–1.53)
Vascular access, *n* (%)
arteriovenous fistulas	17 (81)
central venous catheters	4 (19)
Cause of end-stage renal disease, *n* (%)
glomerulonephritis	6 (28.5)
diabetic renal disease	4 (19.0)
hypertension nephropathy	4 (19.0)
other	7 (33.5)
CCI (point)	4.2±3.1	(range = 2–9)
Estimated GFR (mL/min/1.73 m^2^)	8.9±4.9
Ultrafiltration volume (L)	2.1±1.0	(range = 0.5–3.5)
Hemoglobin (g/dL)	10.4±1.1	(range = 8.0–12.1)
Transferrin saturation (%)	32.5±12.9	(range = 15–58)
Ferritin (ng/mL)	419±375	(range = 35–1622)
Creatinine (mg/dL)	8.02±1.99	(range = 3.9–10.8)
Urea, predialysis (mmol/L)	19.0±4.5	(range = 8.3–28.7)
Urea, postdialysis (mmol/L)	4.9±1.8	(range = 1.5–9.5)
Sodium (mmol/dL)	138.3±1.6	(range = 136–141)
Potassium (mmol/dL)	5.02±0.46	(range = 4.45–5.87)
Albumin (g/L)	39.0±2.1	(range = 35–44)
Phosphorus (inorganic) (mg/dL)	4.86±1.55	(range = 3.0–7.5)
Calcium (mg/dL)	9.0±0.9	(range = 8.1–10.7)
PTH (pg/mL)	320±181	(range = 152–724)

Note: Results are the mean ± standard deviation (SD) or the percentage. Abbreviations: HD, hemodialysis; URR, urea reduction ratio; BFR, blood flow rate; BMI, body mass index; CCI, Charlson Comorbidity Index; GFR, glomerular filtration rate; nPCR, normalized protein catabolic rate; PTH, parathyroid hormone.

## References

[1] Glorieux G., Tattersall J. (2015). Uraemic toxins and new methods to control their accumulation: Game changers for the concept of dialysis adequacy. Clin. Kidney J..

[2] Yamamoto S., Kazama J.J., Wakamatsu T., Takahashi Y., Kaneko Y., Goto S., Narita I. (2016). Removal of uremic toxins by renal replacement therapies: A review of current progress and future perspectives. Ren. Replace. Ther..

[3] Montgomery L.D., Montgomery R.W., Gerth W.A., Lew S.Q., Klein M.D., Stewart J.M., Medow M.S., Velasquez M.T. (2016). Bioimpedance monitoring of cellular hydration during hemodialysis therapy. Hemodial. Int..

[4] Minutolo R., Bellizzi V., Cioffi M., Iodice C., Giannattasio P., Andreucci M., Terracciano V., Di Iorio B.R., Conte G., De Nicola L. (2002). Postdialytic Rebound of Serum Phosphorus: Pathogenetic and Clinical Insights. J. Am. Soc. Nephrol..

[5] Barcellos F.C., Santos I.S., Umpierre D., Bohlke M., Hallal P.C. (2015). Effects of exercise in the whole spectrum of chronic kidney disease: A systematic review. Clin. Kidney J..

[6] Kirkman D.L., Roberts L.D., Kelm M., Wagner J., Jibani M.M., Macdonald J.H. (2013). Interaction between Intradialytic Exercise and Hemodialysis Adequacy. Am. J. Nephrol..

[7] Giannaki C.D., Hadjigeorgiou G.M., Karatzaferi C., Maridaki M.D., Koutedakis Y., Founta P., Tsianas N., Stefanidis I., Sakkas G.K. (2013). A single-blind randomized controlled trial to evaluate the effect of 6 months of progressive aerobic exercise training in patients with uraemic restless legs syndrome. Nephrol. Dial. Transplant..

[8] Hazell T.J., Lemon P.W.R. (2011). Synchronous whole-body vibration increases VO2 during and following acute exercise. Eur. J. Appl. Physiol..

[9] Da Silva-Grigoletto M.E., Vaamonde D.M., Castillo E., Poblador M.S., García-Manso J.M., Lancho J.L. (2009). Acute and Cumulative Effects of Different Times of Recovery From Whole Body Vibration Exposure on Muscle Performance. J. Strength Cond. Res..

[10] Xie L., Jacobson J.M., Choi E.S., Busa B., Donahue L.R., Miller L.M., Rubin C.T., Judex S. (2006). Low-level mechanical vibrations can influence bone resorption and bone formation in the growing skeleton. Bone.

[11] Von Stengel S., Kemmler W., Engelke K., Kalender W.A. (2010). Effect of whole-body vibration on neuromuscular performance and body composition for females 65 years and older: A randomized-controlled trial. Scand. J. Med. Sci. Sports.

[12] Levey A.S., Stevens L.A., Schmid C.H., Zhang Y.L., Castro A.F., Feldman H.I., Kusek J.W., Eggers P., Van Lente F., Greene T. (2009). A New Equation to Estimate Glomerular Filtration Rate. Ann. Intern. Med..

[13] Lowrie E. (1991). The urea reduction ratio (URR): A simple method for evaluating hemodialysis treatment. Contemp. Dial. Nephrol..

[14] Daugirdas J.T. (1993). Second generation logarithmic estimates of single-pool variable volume Kt/V: An analysis of error. J. Am. Soc. Nephrol..

[15] Maduell F., Ramos R., Varas J., Martin-Malo A., Molina M., Pérez-Garcia R., Marcelli D., Moreso F., Aljama P., Merello J.I. (2016). Hemodialysis patients receiving a greater Kt dose than recommended have reduced mortality and hospitalization risk. Kidney Int..

[16] Held P.J., Port F.K., Wolfe R.A., Stannard D.C., Carroll C.E., Daugirdas J.T., Bloembergen W.E., Greer J.W., Hakim R.M. (1996). The dose of hemodialysis and patient mortality. Kidney Int..

[17] Molina Núñez M., Roca Meroño S., de Alcorcon Jimenez R., Hernández G., Jimeno Griño C., Alvarez Fernandez G., Navarro Parreño M., Pérez Silva F. (2010). Kt calculation as a quality indicator of haemodialysis adequacy. Nefrologia.

[18] Locatelli F., Buoncristiani U., Canaud B., Kohler H., Petitclerc T., Zucchelli P. (2004). Dialysis dose and frequency. Nephrol. Dial. Transplant..

[19] Daugirdas J.T., Depner T.A., Inrig J., Mehrotra R., Rocco M.V., Suri R.S., Weiner D.E., Greer N., Ishani A., MacDonald R. (2015). KDOQI Clinical Practice Guideline for Hemodialysis Adequacy: 2015 update. Am. J. Kidney Dis..

[20] Pérez-García R., Jaldo M., Alcázar R., de Sequera P., Albalate M., Puerta M., Ortega M., Ruiz M.C., Corchete E. (2019). Unlike Kt, high Kt/V is associated with greater mortality: The importance of low V. Nefrologia.

[21] Oreopoulos D.G. (2002). Beyond Kt/V: redefining adequacy of dialysis in the 21st century. Int. Urol. Nephrol..

[22] Abbas H.N., Rabbani M.A., Safdar N., Murtaza G., Maria Q., Ahamd A. (2009). Biochemical nutritional parameters and their impact on hemodialysis efficiency. Saudi J. Kidney Dis. Transpl..

[23] Chandrashekar A., Ramakrishnan S., Rangarajan D. (2014). Survival analysis of patients on maintenance hemodialysis. Indian J. Nephrol..

[24] Załuska W., Klinger M., Kusztal M., Lichodziejewska-Niemierko M., Miłkowski A., Stompór T., Sak J., Domański L., Drożdż M., Aksamit D. (2015). Recommendations of the Working Group of the Polish Society of Nephrology for the criteria of quality treatment in dialysis patients with end-stage renal disease. Nefrol. Dial. Pol..

[25] Kosmadakis G., Bevington A., Smith A., Clapp E., Viana J., Bishop N., Feehally J. (2010). Physical Exercise in Patients with Severe Kidney Disease. Nephron Clin. Pract..

[26] Nowicki M., Jagodzińska M., Murlikiewicz K., Niewodniczy M. (2009). Physical activity in dialysed patients—A comparison of different methods of its improvement. Post. Nauk Med..

[27] Smart N., McFarlane J., Cornelissen V. (2013). The Effect of Exercise Therapy on Physical Function, Biochemistry and Dialysis Adequacy in Haemodialysis Patients: A Systematic Review and Meta-Analysis. Open J. Nephrol..

[28] Liao M.T., Liu W.C., Lin F.H., Huang C.F., Chen S.Y., Liu C.C., Lin S.H., Lu K.C., Wu C.C. (2016). Intradialytic aerobic cycling exercise alleviates inflammation and improves endothelial progenitor cell count and bone density in hemodialysis patients. Medicine.

[29] Young H.M.L., March D.S., Graham-Brown M.P.M., Jones A.W., Curtis F., Grantham C.S., Churchward D.R., Highton P., Smith A.C., Singh S.J. (2018). Effects of intradialytic cycling exercise on exercise capacity, quality of life, physical function and cardiovascular measures in adult haemodialysis patients: A systematic review and meta-analysis. Nephrol. Dial. Transplant..

[30] Bohm C., Stewart K., Onyskie-Marcus J., Esliger D., Kriellaars D., Rigatto C. (2014). Effects of intradialytic cycling compared with pedometry on physical function in chronic outpatient hemodialysis: A prospective randomized trial. Nephrol. Dial. Transplant..

[31] Dungey M., Bishop N.C., Young H.M., Burton J.O., Smith A.C. (2015). The Impact of Exercising During Haemodialysis on Blood Pressure, Markers of Cardiac Injury and Systemic Inflammation—Preliminary Results of a Pilot Study. Kidney Blood Press. Res..

[32] Delgado C., Johansen K.L. (2011). Barriers to exercise participation among dialysis patients. Nephrol. Dial. Transplant..

[33] Farese S., Budmiger R., Aregger F., Bergmann I., Frey F.J., Uehlinger D.E. (2008). Effect of Transcutaneous Electrical Muscle Stimulation and Passive Cycling Movements on Blood Pressure and Removal of Urea and Phosphate During Hemodialysis. Am. J. Kidney Dis..

[34] Stenvinkel P., Carrero J.J., von Walden F., Ikizler T.A., Nader G.A. (2015). Muscle wasting in end-stage renal disease promulgates premature death: Established, emerging and potential novel treatment strategies. Nephrol. Dial. Transplant..

[35] Cardinale M., Pope M. (2003). The effects of whole body vibration on humans: Dangerous or advantageous?. Acta Physiol. Hung..

[36] Chanou K., Gerodimos V., Karatrantou K., Jamurtas A. (2012). Whole-body vibration and rehabilitation of chronic diseases: a review of the literature. J. Sports Sci. Med..

[37] Donald Whedon G., Deitrick J.E., Shorr E., Toscani V., Buniak Davis V., Stevens E. (1949). Modification of the effects of immobilization upon metabolic and physiologic functions of normal men by the use of an oscillating bed. Am. J. Med..

[38] Dionello C., Sá-Caputo D., Pereira H., Sousa-Gonçalves C., Maiworm A., Morel D., Moreira-Marconi E., Paineiras-Domingos L., Bemben D., Bernardo-Filho M. (2016). Effects of whole body vibration exercises on bone mineral density of women with postmenopausal osteoporosis without medications: novel findings and literature review. J. Musculoskelet. Neuronal. Interact..

[39] Seefried L., Genest F., Luksche N., Schneider M., Fazeli G., Brandl M., Bahner U., Heidland A. (2017). Efficacy and safety of whole body vibration in maintenance hemodialysis patients—A pilot study. J. Musculoskelet. Neuronal. Interact..

[40] Doyle A., Chalmers K., Chinn D.J., McNeill F., Dall N., Grant C.H. (2017). The utility of whole body vibration exercise in haemodialysis patients: A pilot study. Clin. Kidney J..

[41] Sitja-Rabert M., Rigau D., Fort Vanmeerghaeghe A., Romero-Rodriguez D., Bonastre Subirana M., Bonfill X. (2012). Efficacy of whole body vibration exercise in older people: a systematic review. Disabil. Rehabil..

[42] Iwamoto J., Takeda T., Sato Y., Uzawa M. (2005). Effect of whole-body vibration exercise on lumbar bone mineral density, bone turnover, and chronic back pain in post-menopausal osteoporotic women treated with alendronate. Aging Clin. Exp. Res..

[43] Rees S.S., Murphy A.J., Watsford M.L. (2009). Effects of whole body vibration on postural steadiness in an older population. J. Sci. Med. Sport.

[44] Machado A., García-López D., González-Gallego J., Garatachea N. (2009). Whole-body vibration training increases muscle strength and mass in older women: A randomized-controlled trial. Scand. J. Med. Sci. Sports.

[45] Uehara A., Kurata C., Sugi T., Mikami T., Yamazaki K., Satoh H., Watanabe H., Terada H. (2000). Peak Systolic Blood Pressure in Exercise Testing is Associated With Scintigraphic Severity of Myocardial Ischemia in Patients With Exercise-Induced ST-Segment Depression. Jpn. Circ. J..

[46] Henrique D.M.N., Reboredo M.d.M., Chaoubah A., Paula R.B.d. (2010). Aerobic exercise improves physical capacity in patients under chronic hemodialysis. Arq. Bras. Cardiol..

[47] McGuire S., Horton E.J., Renshaw D., Jimenez A., Krishnan N., McGregor G. (2018). Hemodynamic Instability during Dialysis: The Potential Role of Intradialytic Exercise. BioMed Res. Int..

[48] Madhavan G., Cole J.P., Pierce C.S., McLeod K.J. Reversal of Lower Limb Venous and Lymphatic Pooling by Passive Non-Invasive Calf Muscle Pump Stimulation. Proceedings of the 2006 International Conference of the IEEE Engineering in Medicine and Biology Society.

[49] Fuzari H.K., Dornelas de Andrade A., A Rodrigues M., I Medeiros A., F Pessoa M., Lima A.M., Cerqueira M.S., Marinho P.E. (2018). Whole body vibration improves maximum voluntary isometric contraction of knee extensors in patients with chronic kidney disease: A randomized controlled trial. Physiother. Theory Pract..

[50] Agarwal R. (2012). How can we prevent intradialytic hypotension?. Curr. Opin. Nephrol. Hypertens..

[51] Mueller B.A., Jasiak K.D., Thiel S.R., Stevenson J.M., Vilay A.M., Scoville B.A., Churchwell M.D., Pasko D.A., Perkins N. (2013). Vibration Enhances Clearance of Solutes With Varying Molecular Weights During In Vitro Hemodialysis. ASAIO J..

